# Effect of Departmental and Physician-Selected Interventions on Point-of-Care Ultrasound Documentation Completion

**DOI:** 10.7759/cureus.61675

**Published:** 2024-06-04

**Authors:** Marc I Blatt, Jordan Rupp, Matthew Lipton, Tyler W Barrett, Jeremy S Boyd, Michael J Ward

**Affiliations:** 1 Department of Emergency Medicine, Beth Israel Deaconess Medical Center, Harvard Medical School, Boston, USA; 2 Department of Emergency Medicine, Vanderbilt University Medical Center, Nashville, USA; 3 Department of Emergency Medicine, VA Tennessee Valley Healthcare System, Nashville, USA; 4 Geriatric Research, Education, and Clinical Center, VA Tennessee Valley Healthcare System, Nashville, USA

**Keywords:** interventions, incentives, thematic analysis, survey, semi-structured interviews, mixed-methods design, documentation workflow, point-of-care ultrasound

## Abstract

Background

Point-of-care ultrasound (POCUS) has been disruptive to many experienced emergency physicians as it requires competence in a new physical skill, real-time image interpretation, and navigation of novel software for submission to the electronic health record (EHR). Incomplete documentation of a performed POCUS study used for clinical decision-making represents a potential medicolegal liability, may expose the patient to repetitive or potentially unnecessary imaging, and is a missed opportunity for reimbursement. Identifying effective facilitators of ED POCUS documentation completion requires additional investigation.

Methods

In the first part of this mixed-methods study, eligible attending physicians were stratified into levels of use (“high”/“low”/“never”) based on recent POCUS documentation performance. Semi-structured interviews were conducted with high and low utilizers to explore their perceptions of the POCUS submission workflow and their receptivity to various proposed interventions. Qualitative data were analyzed using a thematic analysis that explored perceived usefulness and usability. The second part of the study consisted of two intervention phases. First, physicians achieving minimum POCUS documentation numbers were rewarded with additional shift scheduling flexibility. In the second phase, the intervention that garnered the most interview support, daily documentation reminder emails, was implemented. The primary outcome was the individual POCUS documentation rates calculated as all studies submitted divided by all studies performed (submitted plus unsubmitted) per month. Provider-level monthly data was aggregated into a departmental rate.

Results

Interviews were conducted with 12 physicians, six from the highest and six from the lowest documentation quartiles. Both groups supported the same two proposed interventions: reminder emails ranked first, then monetary rewards ranked second. High utilizers emphasized the clinical utility of POCUS, whereas low utilizers expressed concerns over “double billing” and exposure to medicolegal liability with uncertain scan interpretations. For low utilizers, a documentation decision could be dependent on the performing resident physician’s displayed confidence. Both groups voiced frustration with the need to use a separate program, Qpath (Telexy Healthcare, Inc, Maple Ridge, British Columbia, Canada), for POCUS documentation. During intervention phase one, the aggregate departmental documentation rate increased from 44.6% to 60.1% with the introduction of the schedule request incentive. This improvement was seen across all documentation quartiles. The departmental rate remained stable and did not improve further following the addition of the daily documentation reminder emails in intervention phase two. When reminder emails ceased yet the day-off request incentive continued, the departmental rate did not drop.

Conclusions

The implementation of a non-financial shift scheduling incentive correlated with the largest increase in departmental POCUS documentation rate. Interviewees incorrectly predicted that email reminders would be the most influential intervention highlighting a mismatch between physician perception and effective drivers of behavior change. Further investigation may focus on determining the size and longevity of the isolated impact of a schedule request incentive, as one might expect diminishing marginal utility.

## Introduction

Point-of-care ultrasound (POCUS) has been used in the care of emergency department (ED) patients for more than 30 years [1-3]. The American College of Emergency Physicians identified POCUS as a fundamental skill that adds value to healthcare delivery in the following ways: improved procedural safety, shortened ED length of stay, superior clinical decision-making, and smarter departmental resource allocation [4]. However, adoption among emergency physicians has been variable with higher uptake in academic EDs [5].

To many experienced emergency physicians who received limited, if any, POCUS training during residency, the introduction of POCUS can be disruptive since they must gain competence in a new physical skill: manipulation of an ultrasound transducer to acquire sufficient images to answer a clinical question. In addition, POCUS requires real-time image interpretation and navigation of novel software to submit ultrasound studies to the electronic health record (EHR). Previous attempts to close this knowledge gap and increase POCUS use include the creation of a fellowship program and the initiation of a faculty training curriculum, among others [6,7].

Incomplete documentation of POCUS studies used for clinical decision-making represents a potential medicolegal liability. These studies and their interpretations are inaccessible to consultants and future providers which may result in repetitive or potentially unnecessary imaging (including ionizing radiation). Further, the hospital does not receive reimbursement for the physician’s time and expertise without prompt documentation of the study’s interpretation.

A prior study identified potential ED POCUS documentation barriers including competing time demands, use of a separate documentation program from the EHR, and difficulty navigating ultrasound machine keystrokes and software [8]. There is some evidence that education and training [9] as well as financial incentives and penalties [10] can modestly increase the documentation rate. This study aims to gain a better understanding of emergency physicians’ perceptions of the POCUS submission workflow to tailor an intervention that specifically addresses uncovered documentation barriers.

This article was previously presented in abstract format as an oral presentation at the 2023 Mediterranean Emergency Medicine Congress on September 8, 2023.

## Materials and methods

Setting and participants

**Figure 1:**
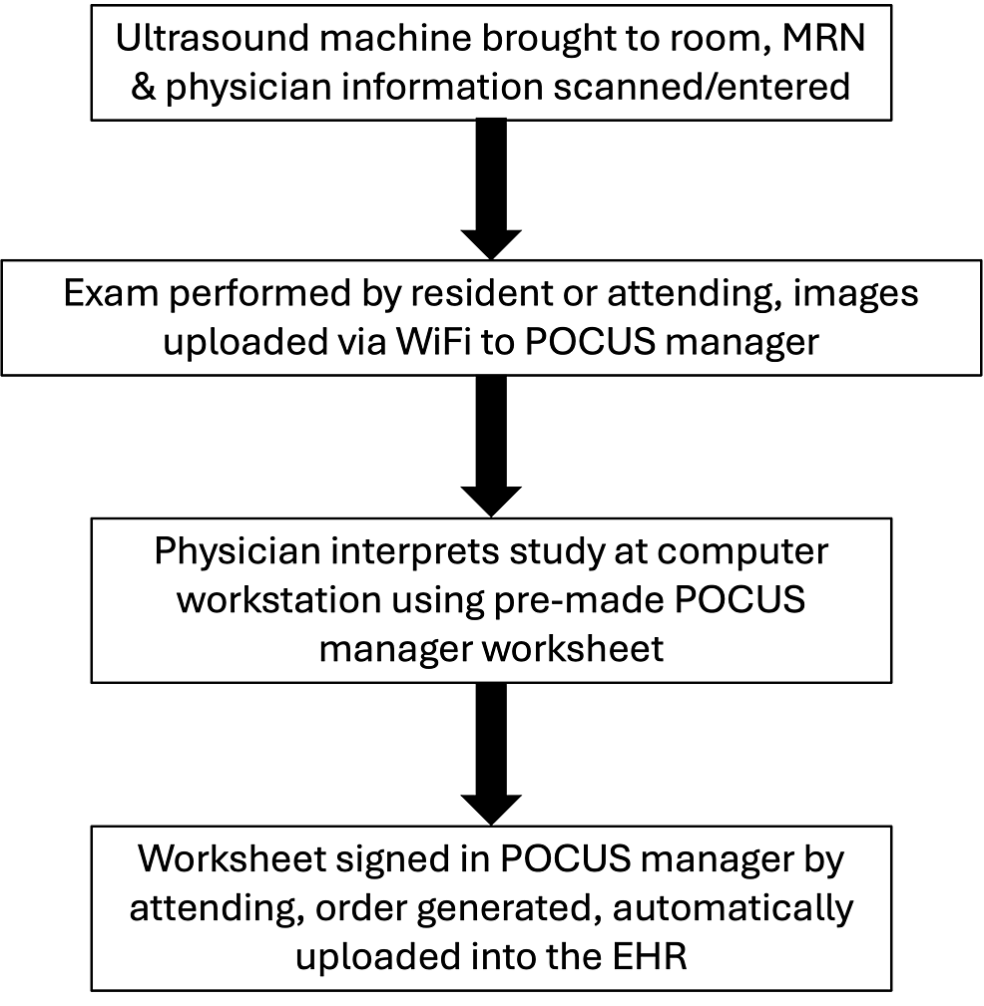
Outline of the documentation workflow for ED point-of-care ultrasound study submission MRN, medical record number; POCUS, point-of-care ultrasound; EHR, electronic health record

The study took place at Vanderbilt University Medical Center in Nashville, Tennessee, United States, a tertiary-care, academic, urban, level I trauma center with an annual ED volume of approximately 70,000 adult patients. Its Department of Emergency Medicine has over 70 faculty members who have wide variability in their comfort level with regular use of POCUS. The department hosts a three-year residency program with 13 trainees per class. Resident POCUS education includes a dedicated two-week rotation during the intern year and continual quality assurance feedback throughout training. The department is constructed with two core clinical pods that are each allocated two ultrasound machines: one Mindray M9 (Mindray Medical International Limited, Shenzhen, Guangdong, China) and one SonoSite X-Porte (FUJIFILM SonoSite, Bothell, Washington, United States). One of the pods is higher acuity and has four trauma bays with a dedicated machine. Additionally, there is a Mindray TE7 housed in the fast-track area. Figure 1 outlines the documentation workflow for a POCUS study completed in the ED. The study institution uses Epic (Epic Systems Corporation, Verona, Wisconsin, United States) for its EHR and Qpath (Telexy Healthcare, Inc, Maple Ridge, British Columbia, Canada) for POCUS manager software/image archiving.

The study included 68 emergency medicine attending physicians who were clinically active for the duration of the study period: July 2020-July 2021. An additional six faculty members with fellowship training in ultrasound were excluded from the analysis because of their unique familiarity with the POCUS submission workflow, as well as their POCUS expertise. Physicians who either stopped working clinically or joined the faculty during the study period were also excluded.

This study was approved by the Institutional Review Board at Vanderbilt University Medical Center (ID number 202525).

Design

This was a mixed-methods study with two parts. In the first part, semi-structured interviews were conducted with high and low utilizers to provide insight into current documentation barriers and to evaluate potential interventions. Eligible physicians were stratified by quartile into “high,” “low,” and “never” utilizers based on recent POCUS documentation performance. A convenience sample of six of the high utilizers and six of the low utilizers were invited to participate in recorded audiovisual interviews (Zoom Video Communications, San Jose, California, United States). Our semi-structured interview guide can be found in Appendix A. All interviews were transcribed by the corresponding author.

The second part of the study involved two intervention phases. The first intervention phase included an incentive program to encourage ED POCUS submission beginning February 1, 2021, at the start of Period 2. This intervention was generated by the ED administrative team. Physicians who correctly documented a minimum number of scans were rewarded with additional schedule flexibility, as they were given additional day-off requests for the next scheduling period.

The second intervention phase of the study implemented an intervention that was selected based on the semi-structured interviews. The survey results of the interviews of both high and low utilizers were tallied to determine the most supported intervention: daily emails to remind physicians to submit their pending ultrasound studies. This intervention was installed at the start of Period 3 and was in addition to the ongoing scheduling incentive by ED leadership. Batches of documentation reminder emails were sent manually once daily every weekday between April 1, 2021, and May 31, 2021. Appendix B contains the reminder email template. A documentation reminder was sent each time an eligible physician was listed as the responsible attending on a saved but unsubmitted POCUS scan within the POCUS management system. The effectiveness of the email intervention and schedule request incentive in augmenting POCUS documentation was assessed through retrospective data analysis. This was accomplished by exporting data from Qpath at the study institution.

Analysis

Quantitative data were analyzed using simple descriptive statistics. In study part one, summary statistics were calculated for each quantitative interview response. In the second part of the study, aggregate ratios were reported by summing provider-level monthly data to calculate a departmental rate. Thematic analysis was performed on the qualitative data using the Technology Acceptance Model (TAM), a theoretical framework that explores how technology is adopted as a function of the technology’s perceived usefulness and ease of use [11,12].

Outcome measures

The primary outcome was the rate of POCUS documentation for each eligible attending physician monthly for the duration of the study period. A physician’s POCUS documentation rate was calculated as the sum of all complete POCUS studies submitted, divided by the sum of all studies performed (submitted plus unsubmitted) per month. We defined a “submitted” study as a POCUS scan with (i) images and/or clips saved to Qpath, (ii) indication and interpretation completed in Qpath, and (iii) signature of the attending physician in Qpath. Completion of these steps resulted in the automatic submission of the study report to the Epic EHR. An “unsubmitted” study was a POCUS scan with images acquired and saved to POCUS manager software without an attending signature, regardless of whether the study was interpreted. The provider-level monthly data was then aggregated for all eligible physicians to calculate a departmental rate: total POCUS studies submitted over total POCUS studies performed.

A secondary outcome measure was calculated for the entire group during Period 3 to help isolate the effect of the daily documentation reminder emails. The emails and associated medical record numbers were stored. The physicians' response to the emails was measured by revisiting the Qpath software to assess documentation completion. The collective impact of the reminder emails was calculated by dividing the number of successfully submitted ultrasound studies in Qpath by the number of unsubmitted ultrasound studies originally included in all emails sent during Period 3. This departmental ratio was reported in aggregate for both April 2021 and May 2021 for all eligible physicians.

## Results

Part one

Of the 68 eligible faculty members without ultrasound fellowship training, 47 physicians completed at least one POCUS study and were included in the initial interviews. Individual POCUS documentation rates were a median of 40% (interquartile range: 26.3%-59.3%). Between February 12, 2021, and March 5, 2021, interviews were performed with six attending physicians in the highest documentation quartile (“high utilizers”) and another six in the lowest documentation quartile (“low utilizers”). Interviewees were not informed of their relative documentation performance.

Survey data collected during the interviews is summarized in Table 1. Both high and low utilizers had a similar number of supervised POCUS scans per shift (1.5 versus 1.3), and both groups had similar comfort levels attesting studies (5.0 versus 4.0). Both groups supported the same two proposed interventions: Qpath-generated reminder emails for unsubmitted studies ranked first, then monetary rewards ranked second. High utilizers valued periodic performance comparisons with their colleagues, whereas low utilizers valued one-on-one training sessions. Both high and low utilizers identified the day-off request incentive as moderately efficacious (3.0 versus 3.0), yet both groups ranked it relatively low in terms of helpfulness (high utilizers 4 versus low utilizers 7).

**Table 1:** Summary statistics of responses to quantitative interview questions for high utilizers and low utilizers POCUS, point-of-care ultrasound; IQR, interquartile range

Survey Component	High Utilizers	Low Utilizers
Reported Average # POCUS Studies Supervised Per Shift	1.5 (1.1,1.9)	1.3 (1.0,1.5)
Likert Scale (Likert 1-5; 1 = no comfort)		
Comfort Level Attesting Saved Study, median (IQR)	5.0 (4.3-5.0)	4.0 (3.3-4.0)
Previous Monetary Incentive Efficacy, median (IQR)	2.5 (2.0-3.0)	2.5 (2.0-3.3)
Current Day-Off Request Incentive Efficacy, median (IQR)	3.0 (2.3-3.8)	3.0 (1.5-3.0)
Proposed Interventions (Ranked Most Helpful (1) to Least Helpful (7))		
Qpath-Generated Reminder Emails	1	1
Group Training Sessions	5	5
1-on-1 Training Sessions	6	3
Monetary Reward	2	2
Resident Physician Engagement	7	6
Additional Day-Off Requests	4	7
Periodic Performance Comparison to Colleagues	3	4

Thematic analysis of the qualitative interview data was performed using the TAM as a guiding theoretical framework. We present identified themes by TAM domain, usefulness and usability, for high utilizers followed by low utilizers.

Perceived Usefulness

POCUS’s role in clinical decision-making was one theme that emerged from our analysis related to the perceived usefulness domain. High utilizers commented on the importance of POCUS for procedural guidance, as well as for the workup of a core set of patient presentations (e.g., undifferentiated hypotension). High utilizers expressed frustration at resident physicians who used an unsupervised POCUS study in their medical decision-making, but then either did not save the images or forgot to notify the responsible attending for attestation. Some high utilizers often felt compelled to repeat the resident’s POCUS study to confirm the findings whenever this took place.

In contrast, some of the low utilizers questioned the necessity of POCUS documentation in certain scenarios. Multiple low utilizers mentioned a fear that the patient or insurance company would be inadvertently “double billed”, the idea that a patient might receive a bill for a POCUS study and then another one for a radiology-performed study used to confirm or rule out a POCUS finding. Similarly, one low utilizer questioned the ethics of charging a patient for a study that does not provide actionable information without confirmation from a different imaging modality.

Even though undocumented POCUS studies are a recognized source of medicolegal liability, low utilizers also worried about exposing themselves to relatively more liability by introducing an uncertain or incorrect POCUS interpretation into the patient’s chart.

Usability

We identified two themes focused on the ease-of-use domain: the role of the resident physician and frustration with the use of a separate POCUS software application. High utilizers felt confident in their interpretation of POCUS images obtained by the resident physicians who they were supervising. Several high utilizers allocated time while on shift to both review images within Qpath and to complete the associated Qpath worksheet with pertinent findings on behalf of the resident.

On the other hand, low utilizers’ documentation decisions were often dependent on the displayed confidence and apparent competence of the resident physician performing the POCUS study. Multiple low utilizers commented on the absence of ultrasound education in their prior residency training. One admitted that they were more likely to sign off on a study if the performing resident appeared confident in their image acquisition and interpretation.

Both high and low utilizers recognized the separation between the EHR and POCUS manager software applications as a documentation impediment. High utilizers focused on the absence of notification of an unsubmitted study. One high utilizer explained the challenges in identifying pending studies; an unsubmitted study requiring attestation could go unnoticed by an attending for a prolonged period of time unless they remembered to log in to Qpath and identify the study by sorting all studies by name. Low utilizers agreed and brought up an additional barrier to documentation, difficulty opening the POCUS manager software on the workstations. One low utilizer found it difficult to locate the Qpath app on the desktop and suggested that the ability to launch Qpath from within Epic would eliminate this obstacle.

Part two

**Figure 2:**
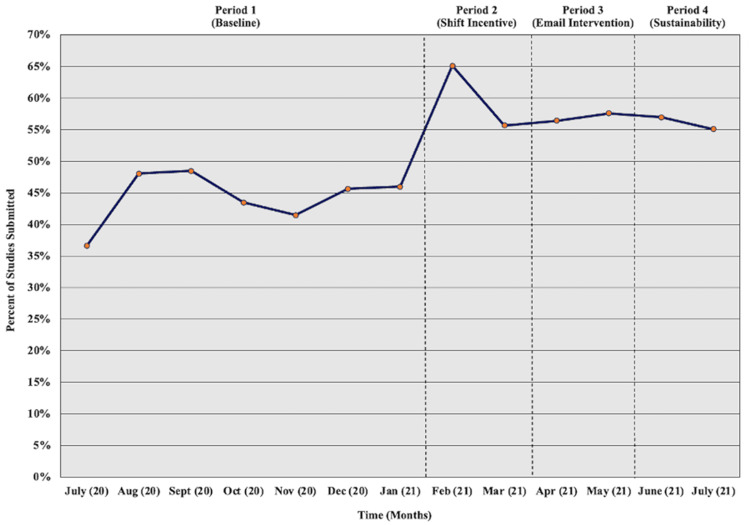
Percent of POCUS studies submitted per month for all eligible physicians combined over the study period (July 2020 – July 2021) POCUS, point-of-care ultrasound

The departmental rate of POCUS documentation, total studies submitted divided by total studies performed for all 68 physicians without POCUS training, is shown in Figure 2 for each month of the study period. This aggregate ED documentation rate increased from 44.6% to 60.1% between the baseline (Period 1) and the shift incentive phase (Period 3). The rate remained stable and did not improve following the addition of daily documentation reminder emails (Period 3). When the reminder emails stopped, but the day-off request incentive continued (sustainability, Period 4), the departmental documentation rate remained level for the next two months without dropping off.

To further isolate the effect of the second study intervention, an additional departmental ratio was calculated. This aggregate email completion ratio was 45.1% in April 2021 (65/144) and 38.2% (50/131) in May 2021.

Table 2 splits all 68 eligible physicians into quartiles based on each physician’s individual POCUS documentation rate, total studies submitted over the total performed, for the duration of the study (all four time periods). The first quartile includes the 17 physicians who submitted POCUS studies at the lowest rate overall, whereas the fourth quartile includes those 17 physicians with the highest overall submission rates. The aggregate monthly data show an absolute increase in documentation rate following the day-off request incentive (Period 2) for all quartiles. This same effect was not seen in Period 3, the second intervention phase. The overall number of POCUS studies performed differed between groups with the first quartile performing only 315 studies compared to 1,211 studies performed by physicians in the fourth quartile.

**Table 2:** Total studies submitted, total studies performed, and documentation rate for all physicians, as well as for quartiles of 17 physicians each based on individual documentation rate over the entire study period

	Period 1 (Baseline)	Period 2 (Shift Incentive)	Period 3 (Email Intervention)	Period 4 (Sustainability)	Overall
All Physicians (N=68)					
Total Submitted	537	353	364	224	1478
Total Performed	1205	587	639	400	2831
Submitted/Performed (%)	45%	60%	57%	56%	52%
First Quartile (Low, N=17)					
Total Submitted	13	12	5	2	32
Total Performed	148	75	61	31	315
Submitted/Performed (%)	9%	16%	8%	7%	10%
Second Quartile (N=17)					
Total Submitted	77	66	47	27	217
Total Performed	293	139	159	76	667
Submitted/Performed (%)	26%	48%	30%	36%	33%
Third Quartile (N=17)					
Total Submitted	67	96	106	65	334
Total Performed	212	145	161	120	638
Submitted/Performed (%)	32%	66%	66%	54%	52%
Fourth Quartile (High, N=17)					
Total Submitted	380	179	206	130	895
Total Performed	552	228	258	173	1211
Submitted/Performed (%)	69%	79%	80%	75%	74%

## Discussion

This mixed-methods study identified three insights into ED POCUS documentation: (i) neither study intervention made a consequential impact on low utilizers, (ii) the intervention that both high and low utilizers had identified as most helpful (reminder emails) did not increase documentation, and (iii) the implementation of a non-financial incentive (shift scheduling flexibility) corresponded to a 15.5% absolute increase in departmental documentation completion with improvement seen in all physician quartiles.

During part one’s semi-structured interviews, key themes were revealed. High utilizers emphasized the clinical utility of POCUS and its central role in medical decision-making, whereas low utilizers expressed concerns over “double billing” and exposure to medicolegal liability with an uncertain scan interpretation. The second part of the study showed a positive correlation between the initiation of an administrative incentive, additional schedule flexibility through day-off requests, and POCUS documentation rate among attending physicians at an academic ED. The proposed intervention that received the most interview support (documentation reminder emails) did not appreciably improve documentation.

At teaching institutions where most ED POCUS scans are performed by residents, attending physicians further from residency training may have had little-to-no supervised practice to build comfort over time. The result is a spectrum of POCUS ability among supervisors with some members of the less-skilled group reticent to sign off on certain study interpretations. A hands-on ultrasound didactic session for faculty that narrowly focuses on the images required for the highest-yield, most-utilized study types could begin to close this knowledge gap. Regarding low utilizers’ double-billing fear, it is largely unfounded, as medical coding and billing specialists are trained for this specific situation, and insurance companies will not reimburse for POCUS in these instances [13].

Both groups shared frustration over the separation between the EHR and POCUS manager software and believed email notifications would serve as a helpful reminder. Several extra mouse clicks and an additional system sign-in represented a formidable on-shift documentation barrier. POCUS manager products, like Qpath, require additional clicks that are known to serve as a workflow completion impediment [14]. Efforts had been made to reduce clicks and simplify the ED POCUS submission workflow (e.g., barcode scanners), but at shift completion, pending studies requiring attestation in Qpath were often inadvertently overlooked. Potential operational improvements include an ED workstation dedicated to POCUS image review and interpretation, streamlined worksheets for each study type, reduced clicks within the POCUS manager, pending study notification automation, and continued faculty ultrasound education.

The implementation of a departmental scheduling incentive correlated with the largest improvement in POCUS documentation rate. The physician-selected study intervention, documentation reminder emails, was then layered on top of the already-existing schedule incentive. During semi-structured interviews, both groups identified day-off requests as a relatively less helpful intervention, yet with its introduction, physician documentation workflow completion increased by over 15%. There was no further improvement with the addition of the intervention that had received the most interview support. This mismatch between perception and behavior change highlights a major administrative challenge: experienced physicians may have poor insight into their own work habits and motivations. Future research efforts could investigate the influence of a physician’s schedule autonomy not just as a “carrot,” but also as a “stick” (for example, by limiting schedule flexibility in response to inadequate documentation).

When participants were split into quartiles based on completion rates, the first and second quartiles had a drop in their documentation rate from Period 2 to Period 3, whereas the third and fourth quartiles continued to document at a similar rate. This may represent a waning of the influence of the schedule request incentive on these physician groups rather than a negative effect from the study intervention. Low utilizers may have felt confident in their ability to submit one study per month to gain one extra day-off request but averaging three submitted studies per month for a second schedule request seemed overambitious. This finding suggests that those documenters in the middle should likely be the target of future campaigns to maximize the incentive’s effect. Efforts expended on performers at either extreme, either never or frequently documented, may be less fruitful.

Regarding incentive implementation, rewards can shape an employee’s behavior, but may unwittingly contribute to his/her burnout [15]. The financial incentivization of patients has created ethical, regulatory, and scalability problems that may also apply when financially rewarding a physician’s desired behavior [16]. Personalized performance feedback and peer comparison between physicians can be equally problematic [17]. That said, non-financial incentives are rarer [18] and may enhance intrinsic motivation, which is thought to be stronger and more durable than a monetary reward [19]. Additional day-off requests do create extra complexity for the schedule coordinator; however, they do not cost the department any additional funds and provide physicians with more autonomy while keeping work hours constant. This incentive was swiftly initiated by the department and has remained sustainable. This study’s results may help guide administrators in designing optimized POCUS submission workflows and incentive structures at other similar institutions.

One limitation of the study was the inability to fully isolate the effect of the reminder email intervention from the ongoing effect of the schedule request incentive. Some physicians did complete ultrasound documentation after email prompting, but the effect of the email reminders on the overall departmental workflow completion appeared nominal. This was further supported by the absence of a drop in the departmental documentation rate during Period 4. Given the risk of confounding, further investigation may focus on determining the size and longevity of the isolated impact of both the schedule request incentive and the reminder emails (either manually or automatically generated).

A potential limitation to this study’s generalizability could be the underlying impact of the COVID-19 pandemic on ED patient volume, relative frequency of chief complaints, and even the ED POCUS workflow. ED providers may have performed relatively less POCUS to limit viral exposure and/or the need for equipment decontamination. Lastly, the study was performed at a single academic site, so the results may not be generalizable to community or county emergency departments, especially those without training programs.

## Conclusions

The implementation of a non-financial shift scheduling incentive correlated with the largest increase in departmental POCUS documentation rate. Interviewees incorrectly predicted that email reminders would be the most influential intervention highlighting a mismatch between physician perception and effective drivers of behavior change. Further investigation may focus on determining the size and longevity of the isolated impact of a schedule request incentive, as one might expect diminishing marginal utility.

## Figures and Tables

**Figure 3:**
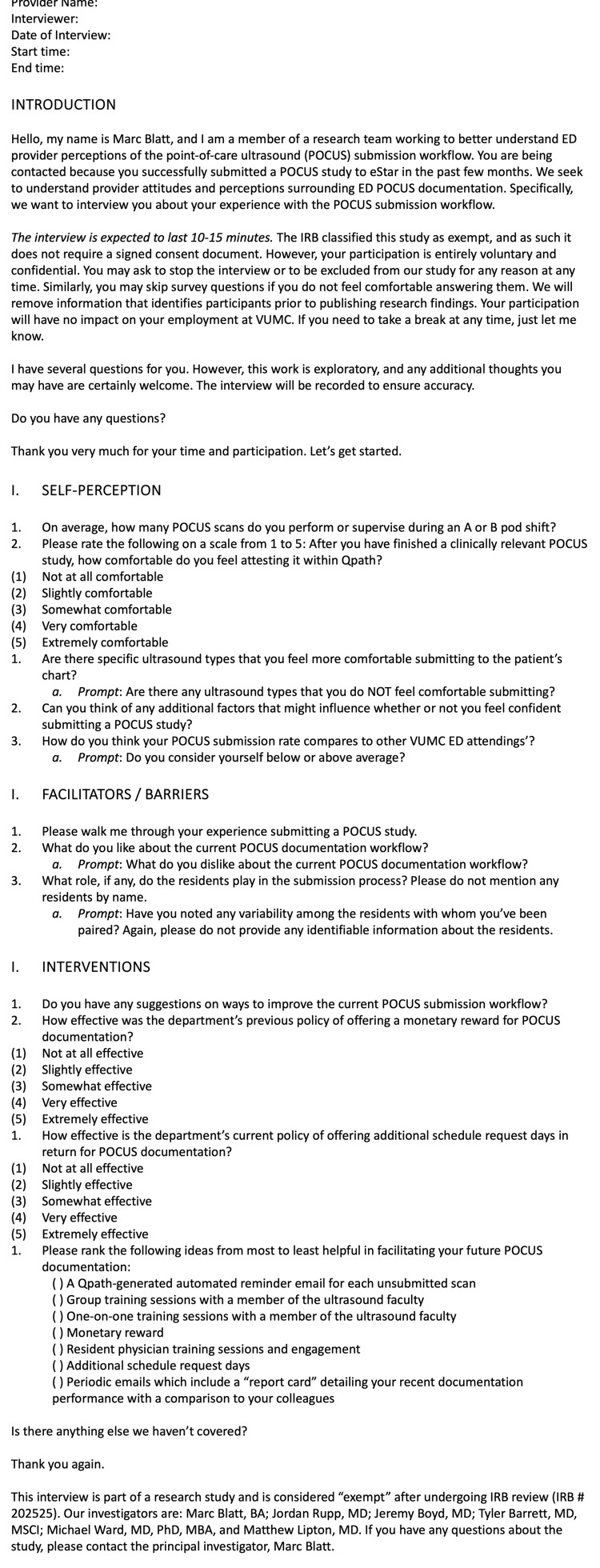
Semi-structured interview guide

**Figure 4:**
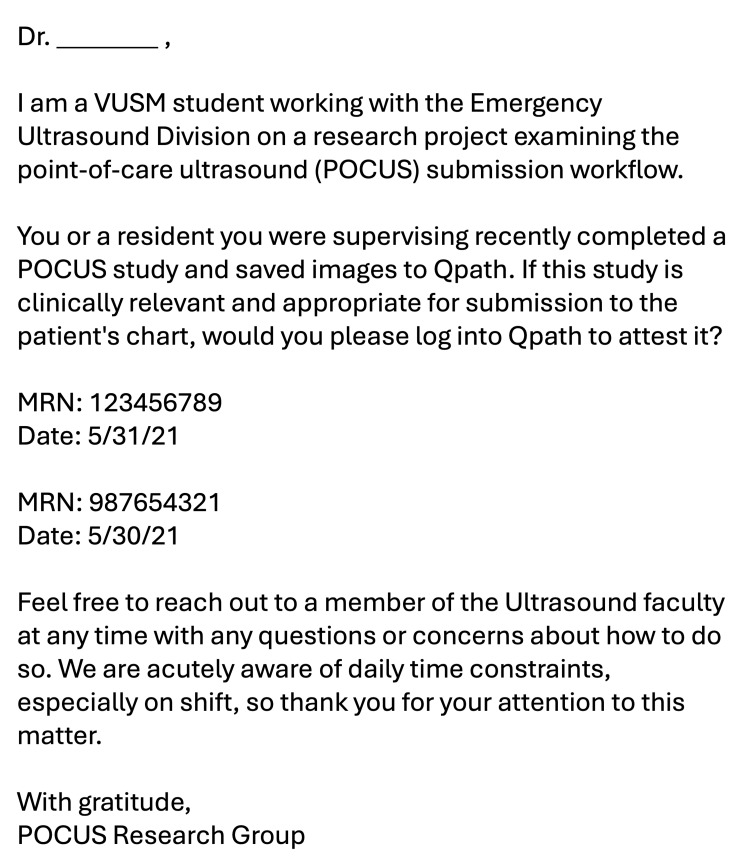
Reminder email template

## Appendices

Appendix A

Appendix B
